# Collembolan Transcriptomes Highlight Molecular Evolution of Hexapods and Provide Clues on the Adaptation to Terrestrial Life

**DOI:** 10.1371/journal.pone.0130600

**Published:** 2015-06-15

**Authors:** A. Faddeeva, R. A. Studer, K. Kraaijeveld, D. Sie, B. Ylstra, J. Mariën, H. J. M. op den Camp, E. Datema, J. T. den Dunnen, N. M. van Straalen, D. Roelofs

**Affiliations:** 1 Department of Ecological Science, VU University Amsterdam, Amsterdam, The Netherlands; 2 EMBL-European Bioinformatics Institute, Cambridge, United Kingdom; 3 Microarray Facility, VU Medical Center, Amsterdam, The Netherlands; 4 Department of Microbiology, Radboud University Nijmegen, Nijmegen, The Netherlands; 5 Keygene NV, Wageningen, The Netherlands; 6 Leiden Genome Technology Center, Human and Clinical Genetics, Leiden University Medical Center, Leiden, The Netherlands; Max F. Perutz Laboratories, AUSTRIA

## Abstract

**Background:**

Collembola (springtails) represent a soil-living lineage of hexapods in between insects and crustaceans. Consequently, their genomes may hold key information on the early processes leading to evolution of Hexapoda from a crustacean ancestor.

**Method:**

We assembled and annotated transcriptomes of the Collembola *Folsomia candida* and *Orchesella cincta*, and performed comparative analysis with protein-coding gene sequences of three crustaceans and three insects to identify adaptive signatures associated with the evolution of hexapods within the pancrustacean clade.

**Results:**

Assembly of the springtail transcriptomes resulted in 37,730 transcripts with predicted open reading frames for *F*. *candida* and 32,154 for *O*. *cincta*, of which 34.2% were functionally annotated for *F*. *candida* and 38.4% for *O*. *cincta*. Subsequently, we predicted orthologous clusters among eight species and applied the branch-site test to detect episodic positive selection in the Hexapoda and Collembola lineages. A subset of 250 genes showed significant positive selection along the Hexapoda branch and 57 in the Collembola lineage. Gene Ontology categories enriched in these genes include metabolism, stress response (i.e. DNA repair, immune response), ion transport, ATP metabolism, regulation and development-related processes (i.e. eye development, neurological development).

**Conclusions:**

We suggest that the identified gene families represent processes that have played a key role in the divergence of hexapods within the pancrustacean clade that eventually evolved into the most species-rich group of all animals, the hexapods. Furthermore, some adaptive signatures in collembolans may provide valuable clues to understand evolution of hexapods on land.

## Introduction

The Hexapoda represent a monophyletic lineage within the Pancrustacea [1–3]. This lineage includes the insects and a number of apterygote groups such as Collembola, Diplura, Protura and Archaeognatha. Recent phylogenetic analysis has shown that Hexapoda represent one of six terrestrialization events within the Ecdysozoa [4]. According to this reconstruction, Hexapoda derive from a crustacean ancestor. This is supported by a study from Von Reumont et al., which suggests that hexapods most likely derive from a single terrestrialization event of an originally crustacean lineage [5].

*Folsomia candida* and *Orchesella cincta* are members of the hexapod subclass Collembola (springtails). They are common and widespread distributed arthropods that inhabit soil, leaf litter and other decaying plant material, where they function as decomposers of organic matter driving mineralization [6]. The springtail *F*. *candida* reproduces parthenogenetically at a high rate, which makes it a suitable model for laboratory experimentation, including bioassays for soil contamination [7, 8]. *Orchesella cincta* is a sexually reproducing collembolan living in the litter layer rather than inside the soil. It shows a wide ecological distribution including human-disturbed areas and shows a remarkable genetic variability and potential for stress tolerance evolution [9].

From an evolutionary point of view, Collembola are intriguing, since they share the most recent common ancestor with insects [1, 3, 10]. With a total of about 8000 species they represent a widespread and abundant group of terrestrial arthropods worldwide [11]. Their body plans lack some insect features such as wings, malpighian tubules and cessation of moulting in the adult stage. Collembolans evolved morphological features that are unusual for hexapods, such as a furcula, used to jump, and a ventral tube that plays a role in the water balance and osmoregulation [12–14]. The possession of appendages, such as the furcula, on abdominal segments in Collembola may be seen as a crustacean heritage; however, the abdominal appendages of Crustacea are less specialized. In contrast, insects lack any abdominal appendages in the adult stage, except cerci in some groups.

It is now commonly accepted that hexapods emerged within the pancrustacean group that evolved on land [1, 5]. Since Collembola and other apterygotes, such as Protura, are at the base of the hexapods clade, it is often suggested that the collembolan divergence coincides with adaptation to diverse terrestrial ecosystems [15]. As such, they may provide key insights in the terrestrialization process of the hexapod animal clade. Collembolans most likely have an edaphic origin, and not an aquatic origin. However, several species of Collembola have a semi-aquatic lifestyle. These have to be considered as secondary adaptations among more derived Collembola [15]. The ancestral position of Collembola is confirmed by fossil evidence. The oldest hexapod fossil is the collembolan *Rhyniella praecursor* dated from the early Devonian, about 400 million years ago [16]. The estimated evolutionary distance between hexapods and crustaceans is 479 million years ago (Mya) and 406 Mya between springtails and insects [1].

Adaptive changes may be caused by various mechanisms of gene evolution. Some studies suggest that changes in gene expression often result in adaptive evolution of regulatory sequences [17]. However, changes in coding sequence most certainly lead to evolutionary transitions as well [18–20]. Another mechanism of evolution is expansion or contraction of gene families. It is suggested that gene gain or loss is a major source of novel gene functions and evolutionary innovation, since it provides opportunities for specific adaptations [21–23]. Although most new genes originate by duplication of preexisting genes [24, 25], new protein-coding genes could also evolve *de novo* out of non-coding sequences [25, 26].

Several studies employed a comparative genomics approach to reveal adaptations associated with key evolutionary transitions. Among them, Ometto et al. analyzed signatures of altitudinal adaptations in brassicaceous plants by comparing the transcriptomes of two *Cardamine* species with the closely related model *Arabidopsis thaliana* [27]. Rands *et al*. compared the genome of the Galapagos ground finch *Geospiza magnirostris*, to the closely related zebra finch and the remotely related chicken, to shed light on the evolution of beak morphology [28]. In both studies genes associated with habitat preference were identified to be under positive selection.

In this study we aim to reveal potential adaptive signatures of Hexapoda evolution. Such signatures could shed new light on the adaptations associated with six-legged arthropods. For this purpose, the protein-coding gene sequences of three insects, three crustaceans and transcriptomes of two springtails were studied. Codon alignments of orthologous clusters were constructed in order to perform likelihood tests on non-synonymous over synonymous ratios to identify genes under positive selection in the Hexapoda and in the Collembola lineages. The genes and gene ontology (GO) categories associated with these processes are discussed in the evolutionary context of these species.

## Materials and Methods

### Transcriptome sequencing and assembly

To obtain complete transcriptomes multiple stress treatments were applied on several important developmental stages (eggs, juveniles and adults) for *F*. *candida* and *O*. *cincta*. The following stress treatments were included: heat exposures to 10°C, 20°C, 30°C and 35°C for 3 days; desiccation treatment for 4 days in Lufa standard soil at 20% of water holding capacity; pH treatment at pH 3 for 4 days; toxicity exposures to cadmium and phenanthrene according to ISO guidelines [8] at the EC50 (concentration causing 50% decrease in reproduction) for 3 days. Even though large numbers of genes were transcriptionally activated, no mortality in adults was observed at such EC50 levels in previous studies [29, 30]. Animals were immediately snap frozen in liquid nitrogen and stored at -80°C to guarantee RNA integrity. Each developmental stage and treatment was replicated twice. For each species the final RNA pool was represented by 12 μg of total RNA, prepared by mixing 500 ng of each of the 24 RNA samples.

1.5 μg of normalized cDNA in 100 μl 10 mM Tris/HCl was fragmented. cDNA normalization was performed by Evrogen [31, 32]. After fragmentation, end repair, and ligation of adapters, enrichment of the 150–250 base pairs (bp) fragments was done using the Paired-End Sequencing kit of Illumina, according to the manufacturer’s protocol. All purification steps were done using the MinElute PCR purification kit (Qiagen). Quality and concentration of the fragmented and enriched library was verified on a lab-on-a-chip (Agilent Technologies).

Next-generation sequencing (NGS) of the 150–250 bp fragments for *F*. *candida* and *O*. *cincta* was performed on the Next Genome Analyzer II platform and on the Illumina HiSeq 2000 platform (Illumina, Inc.), respectively. The sequencing data was deposited to NCBI’s Sequence Read Archive (SRA) under accession numbers SRR935329 and SRR935330.

Pre-processing of NGS data was performed using Trimmomatic.0.20 with the recommended parameters [33]. This removed adapters and other Illumina-specific sequences, regions with average quality below 15 within a 4-base wide sliding window, bases below quality 3, N bases from the start and end of the read, and reads that were shorter than 35 bp. Reads were quality assessed with the quality assessment software FastQC [34]. Because *Saccharomyces cerevisiae* (*S*. *cerevisiae*) is the main food source of lab-reared springtails, we expected that the sequences could be contaminated with yeast RNA. Also, contamination by human DNA and by DNA from *Wolbachia*, an obligate bacterial endosymbiont of *F*. *candida* were considered. To check for potential contamination in both datasets, the raw reads were mapped to the genomes of *Homo sapiens* [35], *S*. *cerevisiae* [35] and *Wolbachia* [36] with TopHat 2.0.8 [37] using default parameters. The raw reads that were not mapped to the above genomes were used for the assembly.

Assembly of the *F*. *candida* and *O*. *cincta* transcriptomes was performed with the Trinity package [38]. To reduce the number of isoforms in Trinity transcripts, we conducted additional CAP3 assembly [39]. In order to complement the NGS-derived transcriptomes Expressed Sequence Tags (ESTs) for *F*. *candida* and *O*. *cincta* were downloaded from Collembase [9, 40] together with all publicly available ESTs from the NCBI databases. Subsequently, a CAP3 assembly was performed to merge the ESTs with the assembled transcriptomes where possible. Contigs with length less than 200 bp were identified and excluded from further analyses. Finally, we screened assembled contigs against the UniVec database [41] in order to remove all possible vector contamination. Assembled transcriptomes were submitted to NCBI transcriptome shotgun assembly database (TSA) under BioProject No. PRJNA211850 and PRJNA211851.

The *de novo* assembly quality assessment metrics were proposed by Martin and Wang [42] and were applied to our assemblies as follows. The accuracy and contiguity of the transcriptomes were calculated based on reference EST datasets for both *F*. *candida* and *O*. *cincta*. The accuracy metric is defined as the percentage of the correctly assembled bases estimated using the set of expressed reference transcripts. The contiguity metric is defined as the percentage of expressed reference transcripts covered by a single, longest-assembled transcript. Contiguity was estimated as the percentage of ESTs covered at least on 70% by best Blast hit transcript. Additionally, we performed tBlastN [43] searches of the 248 core eukaryotic genes [44] in the assembled transcriptomes with expect values (e-value) of less than 10^–20^. Finally, we tested for predicted open reading frames (ORFs) using OrfPredictor [45]. Only sequences with a predicted ORF were retained for functional annotation.

### Functional annotation

Functional annotation of the transcriptomes of *F*. *candida* and *O*. *cincta* was performed using the Blast2GO suite [46]. BlastX [43] searches of nucleotide query sequences translated in all reading frames were performed against a non-redundant (nr) protein sequence database [47] with an e-value cut-off of 10^–3^. Top hits with the best alignment and the lowest e-value were selected for each contig. Gene Ontology (GO) terms associated to the hits obtained after a BLAST search were retrieved. GO annotation was executed using the following settings: terms with e-value < 10^–6^, annotation cut-off 50 and GO-Weight of 5. Functional annotation information was obtained from InterPro databases [48] using the InterProScan [49] option in Blast2GO.

Since the largest number of top Blast hits was achieved with *Daphnia pulex (D*. *pulex)* both for *F*. *candida* and *O*. *cincta*, the complete genome of *D*. *pulex* was selected for further comparative analysis. According to several published phylogenies *Daphnia* is one of the most closely related outgroups to springtails and insects [3, 10, 50]. Furthermore, we used protein-coding gene sequences and corresponding amino acid sequences of two other crustaceans *Litopenaeus vannamei (L*. *vannamei)* and *Penaeus monodon (P*. *monodon)* from EvidentialGene [51], and three insects, *Pediculus humanus* (*P*. *humanus*), *Tribolium castaneum* (*T*. *castaneum*) and *Acyrthosiphon pisum* (*A*. *pisum*) from Ensembl Metazoa database [52] as input for comparative analysis.

### Signatures of positive selection

**Fig 1:**
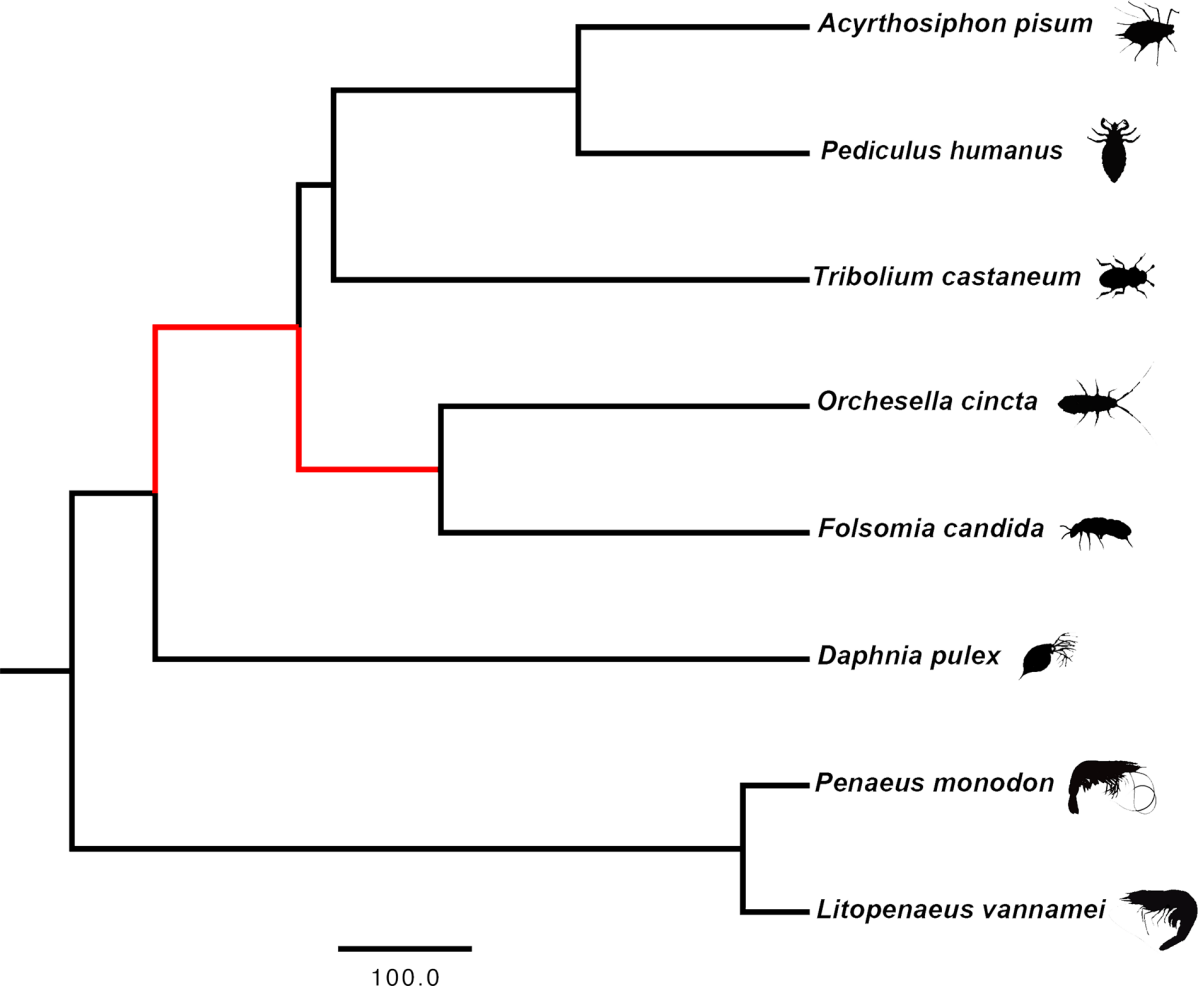
Phylogenetic relationship among *F*. *candida*, *O*. *cincta*, *L*. *vannamei*, *P*. *monodon*, *D*. *pulex*, *T*. *castaneum*, *P*. *humanus and A*. *pisum*. The Collembola lineage is represented by *F*. *candida* and *O*. *cincta*. The Crustacea lineage is represented by two decapods (*L*. *vannamei* and *P*. *monodon*) and a cladoceran (*D*. *pulex*). The Insecta clade is represented by *T*. *castaneum*, *P*. *humanusa* and *A*. *pisum*. The red branches indicate terrestrialization.

Orthologous clusters shared among protein sequences of *D*. *pulex*, *L*. *vannamei*, *P*. *monodon*, *T*. *castaneum*, *P*. *humanus*, *A*. *pisum* and translated transcripts of *F*. *candida* and *O*. *cincta* were predicted by OrthoMCL version 1.4 [53] with default parameters. Single-copy and multiple-copy orthologous protein clusters representing all eight species were examined to identify genes under positive selection. In case clusters contained more than one gene per species, only a single sequence per species with the highest average similarity was selected using T-Coffee version 8.14–1 [54] according to Wissler et al. [55]. Orthologous sequences were aligned with the PRANK algorithm, which considers insertions and deletions as evolutionary events [56]. Protein alignments were then converted back into nucleotide alignments with PAL2NAL v.14 [57]. Alignments with length more than 150 bp without gaps were further analyzed. Columns in the alignment with at least 60% of residues were retained for the analysis. A test for positive selection was performed for each orthologous cluster in CODEML within the PAML package v. 4.7, [58] in order to describe levels of coding sequence evolution and to identify genes undergoing accelerated divergence. The phylogenetic relationships of the eight selected species was used as a reference tree (Fig 1) in this analysis. The Hexapoda branch and the Collembola branch were taken as foreground, while *D*. *pulex* and the two shrimps were used as outgroups to the Hexapoda lineage [10, 59, 60]. To detect positive selection affecting sites along the branch of interest (Hexapoda or Collembola) the branch-site model A from CODEML was applied (options model = 2 and NSsites = 2) [61, 62] for each orthologous cluster. The test uses the ratio (ω = *d*_N_/*d*_S_) of the rate of nonsynonymous (*d*_N_) nucleotide substitutions to that of synonymous (*d*_S_) substitutions between homologous protein-coding gene sequences. Null and alternative models were compared in a likelihood ratio test (LRT) with one degree of freedom. The null model assumes only neutral evolution (ω = 1) in the foreground branch, while the alternative model allows positive selection (ω ≥ 1) in the foreground branch. To avoid false discoveries we corrected *p*-values with a false discovery rate (FDR) cut-off value of 0.05 in the QVALUE package in R [63]. Yang and Dos Reis suggested that the branch-site model could in some cases lead to convergence problems in the log-likelihood calculations, resulting in negative or artificially large log-likelihood values [64]. Therefore, we repeated every run three times to avoid any false positives due to the non-convergence of the null model and any false negatives due to the non-convergence of the alternative model. The largest likelihood values for the null and alternative models were retained. Negative likelihood ratio values were assumed to be estimates of zero.

We applied Blast2GO [46] with default parameters to the protein sequences of each species to characterize them with the GO terms and sequence annotations. We functionally annotated clusters by assigning GO terms associated with genes within an orthologous group. Subsequently, genes under positive selection in the Hexapoda clade and in the Collembola clade were tested for the enriched GO terms against all clusters analyzed for the positive selection using the elim algorithm in the topGO package [65] in R (version 3.1.2.). This algorithm computes the *p*-value of a GO term and removes the genes annotated to significant GO terms from all ancestors of these GO terms. The elim algorithm reduces false-positive rate, but with a risk of discarding relevant nodes [66]. Notably, the elim test is not independent since the *p*-value of a GO term relies on the neighboring terms. Consequently, multiple testing theory does not directly apply [65]. Gene Ontology terms related to biological processes and molecular functions with more than one gene present in the reference and test sets we considered to be significantly enriched at *p*-value less than 0.05.

## Results and Discussion

### Transcriptome sequencing and assembly

In total 88,385,689 paired-end reads were generated for *F*. *candida* and 18,994,903 paired-end reads for *O*. *cincta*. The pre-processing step eliminated very short and low-quality fragments: 15.1% of *F*. *candida* reads and 2.8% of *O*. *cincta* reads were omitted (S1 Table). S1 Table further summarizes the number of reads regarded as potential contamination.

The transcriptomes of *F*. *candida* and *O*. *cincta* were successfully assembled *de novo* employing Trinity (Table 1, S1 Fig). After merging the assembled contigs with available ESTs we retrieved 38,015 transcripts for *F*. *candida* and 32,432 transcripts for *O*. *cincta* that exceeded a length of 200 bp. Quality and biological relevance of the assembly was ascertained by applying the quality metrics proposed by Martin and Wang [42]. Table 1 summarizes the statistics for accuracy, contiguity and completeness of assembled contigs for *F*. *candida* and *O*. *cincta* based on the EST reference datasets as well as percentages for the presence of core genes considered essential for all eukaryotes [44]. As much as 99.2% of the eukaryotic core proteins are present in *F*. *candida* and 97.6% are identified in *O*. *cincta*. A subset of 82.3% of core proteins identified in *F*. *candida* was nearly full-length with alignment coverage higher than 70%, while 81% of core proteins were nearly full-length covered in *O*. *cincta*.

**Table 1:** Descriptive metrics of the *F*. *candida* and *O*. *cincta de novo* assembled transcriptomes.

	*F*. *candida*	*O*. *cincta*
Number of contigs	38,015	32,432
N50 (bp)	1,161	1,105
Maximum contig size (bp)	6,069	8,299
Total (bp)	29,540,912	22,484,011
% ESTs with BlastN hit in dataset	99.4	96.6
Accuracy (%)	99.9	99.4
% EST covered more than 70% by best-hit transcript	99.2	95.5
% Core eukaryotic proteins with tBlastN hit in dataset	99.2	97.6
% Transcripts with predicted ORF	99.3	99.1

Table 1 shows that more than 96% of expressed sequence tags (ESTs) matched Trinity-assembled contigs, while at least 95% were aligned on Trinity assembled contigs with coverage of more than 70%. An accuracy metric was calculated as the percentage of correctly assembled bases when compared to the reference ESTs from NCBI. This accuracy was more than 99% for both transcriptomes. Overall, these descriptive metrics indicate that the assemblies are of high quality. The quality control steps also revealed that the two assemblies have comparable quality, despite the fact that two different sequencing platforms (Illumina HiSeq2000 and Illumina GAII) were applied. Finally, we predicted open reading frames (ORFs) in nearly all *de novo* assembled transcripts (Table 1).

### Functional annotation

**Fig 2:**
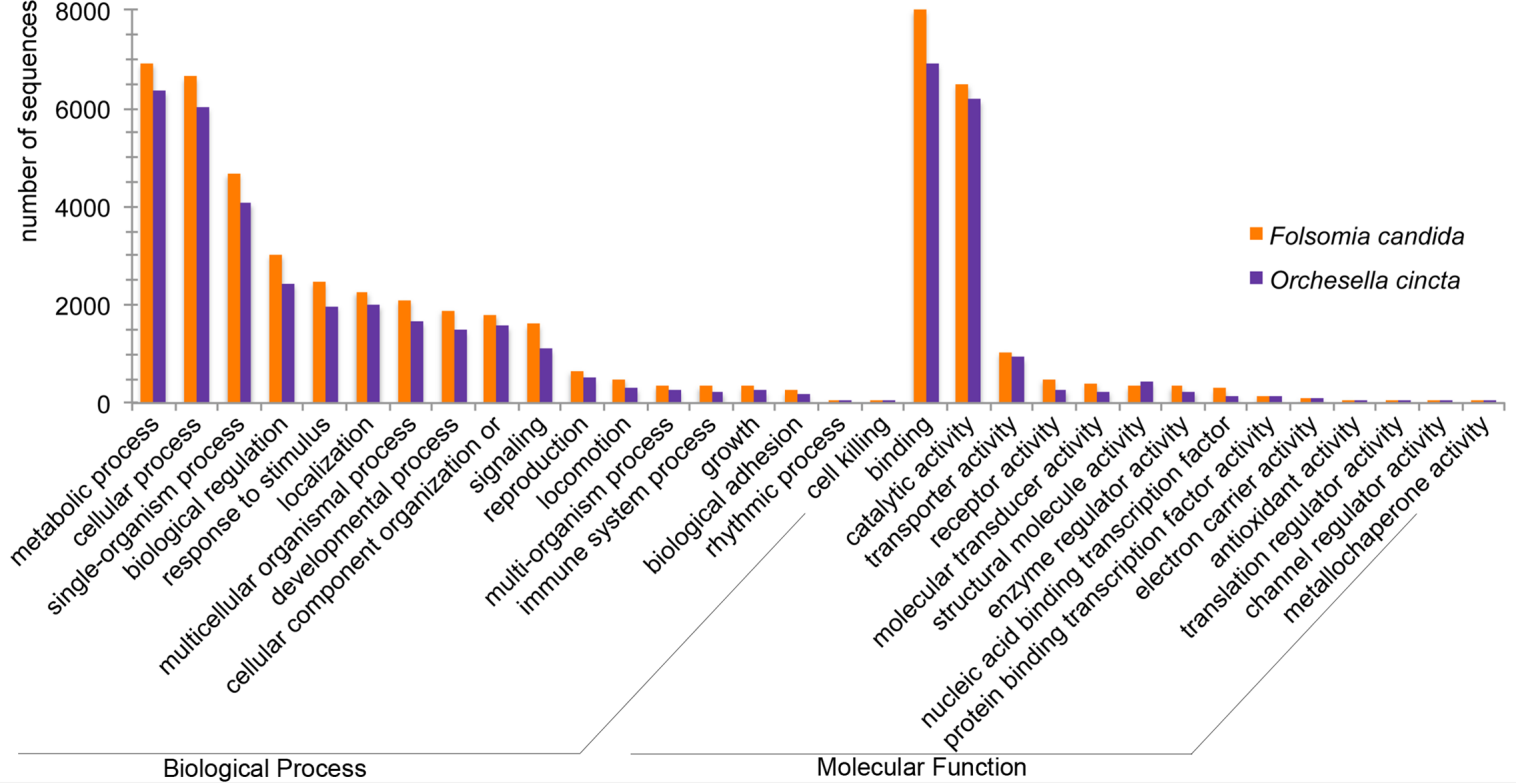
Gene Ontology (GO) distribution in *F*. *candida* and *O*. *cincta* transcriptomes. The bar chart describes the number of genes assigned to the GO biological processes and molecular functions on level 2 with sequence cut-off of 2 sequences.

Annotation with Blast2GO suite [46] resulted in significant BlastX hits to sequences in the non-redundant (nr) database of NCBI for in 44.6% of *F*. *candida* transcripts, while functional annotations could be retrieved for 34.2%. These percentages were slightly higher for *O*. *cincta*, where 48.5% of transcripts showed a significant BlastX hit, and 38.4% could be annotated. The relatively low functional annotation levels for both species can be explained by the lack of full-length sequences and by the relatively large phylogenetic distance between springtails and other genomic model organisms. Fig 2 provides a general overview of the most represented biological processes and molecular functions in both collembolan transcriptomes. Notably, most of the Blast top hits were retrieved from *Daphnia pulex*, followed by *Tribolium castaneum* and *Pediculus humanus* for both collembolan transcriptomes (S2 Fig). Interestingly, this shows that *Daphnia*, a crustacean, rather than the well-investigated genomes of insects such as *Drosophila* and *Tribolium*, produced the highest number of Blast hits with collembolan transcripts. This suggests that transcriptomes of springtails have more genes in common with crustaceans than with insects, although Collembola seem to be more related to Insecta, since they share the six-legged body plan as well as a terrestrial life-style in most cases. We have to note that the high level of similarity between *Daphnia* and Collembola could also be caused by the comparably fast evolution rates in *Tribolium* and *Drosophila*, which have been well documented [67, 68]. Nevertheless, Collembola represent one of the first lineages splitting off after the terrestrialization, still bearing a clear genomic signature of the crustaceans.

To verify whether unknown transcripts in collembolan transcriptomes are species-specific, we performed tBlastX analysis [43] of these unknowns against the other springtail transcriptome and twenty collembolan transcriptomes from 1KITE project [1, 69]. As a result, we determined that 9,820 sequences for *O*. *cincta* (30.1%) and 16,565 (43.6%) sequences for *F*. *candida* do not show a Blast hit. These transcripts could be specific to particular developmental stage or treatment, so they may not be expressed in other transcriptomes. Alternatively, these transcripts could also correspond to the species-specific and strains-specific genes. Such genes are often called orphans, because they lack homology with any other species. They have shown to be a universal feature in genomes and are most likely associated with developmental adaptations and interactions with the environment [26].

Finally, the annotation analysis identified 739 unique enzyme codes associated with *F*. *candida* contigs and 668 enzyme codes associated with *O*. *cincta* contigs. Plotting these codes onto metabolic pathways in iPATH 2.0 [70], indicated that the majority of genes involved in essential metabolic pathways are present in both transcriptomes (S3 Fig). For both organisms the best-represented KEGG pathways are ‘Purine metabolism’, ‘Pyrimidine metabolism’ and ‘Oxidative phosphorylation’.

### Signatures of positive selection

We predicted 22,121 OrthoMCL orthologous clusters shared among species (S2 Table) and detected episodic positive selection acting on genes across the Collembola and the Hexapoda lineages using the branch-site model [61, 62, 64]. Episodic positive selection denotes events that occurred in the past and were preserved by purifying selection. The branch-site model discriminates between amino acid substitutions that have been fixed by genetic drift compared to potentially adaptive substitutions that have been fixed under positive selection. This model divides amino acids in four categories between two groups of species, which are separated by the branch of interest (the foreground branch) [62]. The first category is defined by the amino acids that are under purifying selection; they are conserved between the two groups of species and they are likely to be important for the function of the protein. The second category includes amino acids that are not conserved between two groups and are under relaxed selective pressure. The amino acids in this category are less likely to be important for protein function. The third and fourth categories are represented by the amino acids conserved in one group, but different from the other group of species, they can be either conserved or under a relaxed pattern of substitution. This category is likely to be important for a gain-of-function in a specific clade. By applying a likelihood ratio test to branch-site model we distinguish between neutral evolution and positive selection [62]. The branch-site model has the statistical power to detect ancient adaptive genetic events that could be linked with other biological events, in our case the transition from water to land [71]. A potential problem in these statistical tests is the saturation at the *d*_S_ level over long evolutionary times that can bias the estimated *d*_N_/*d*_S_ ratio. However, recent studies have demonstrated that the branch-site model is robust and conservative, even with long divergence times [72, 73]. These studies used a dataset similar in scale and time divergence to the dataset used in this study. Therefore, the branch-site model is adequate to our usage.

We identified orthologous clusters represented by all eight species and estimated corresponding evolutionary rates. The 2244 orthologous clusters shared among collembolans, crustaceans and insects, were analyzed for positive selection in the Hexapoda and in the Collembola lineages. They include 1128 single-copy gene clusters and 1116 multiple paralog clusters. Selective pressure on each gene along the species tree (Fig 1) was determined by estimating the ratios between non-synonymous versus synonymous substitutions in the coding sequence (*d*_N_/*d*_S_) using a likelihood ratio test (LRT). Firstly, single copy orthologous clusters were considered, since this group is assumed to contain orthologs with the highest confidence [21, 74]. The LRT analysis with false discovery rate correction at 5% revealed 99 cases where the branch-site test for positive selection was significant in the foreground Hexapoda branch and 15 cases with positive selection in the Collembola branch (S3 Table). A less stringent analysis of clusters with multiple paralogs yielded 151 positively selected gene (PSG) cases, whereas 42 PSGs were identified along the Collembolan clade (S3 Table).

**Fig 3:**
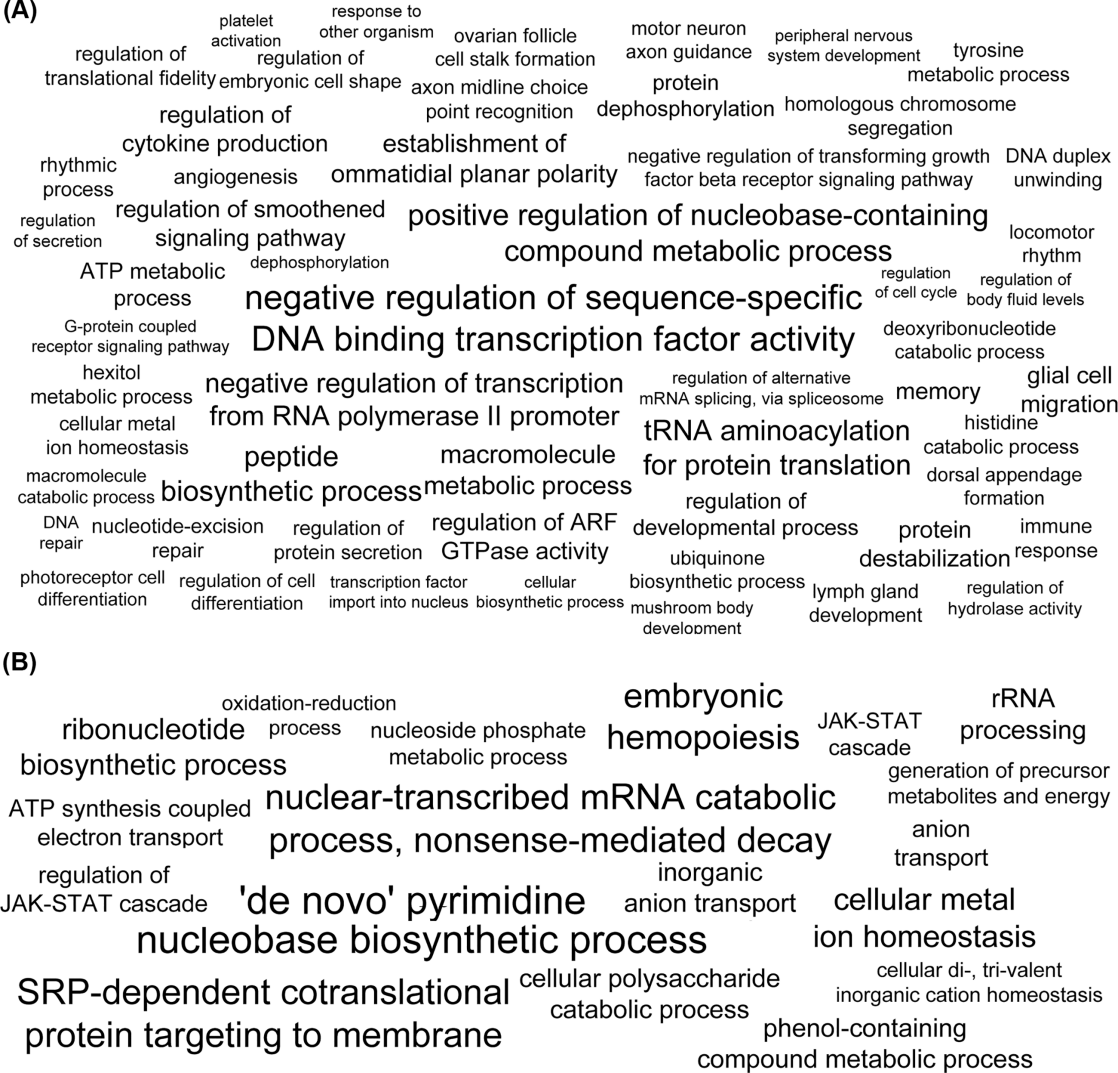
Term cloud of over-represented Gene Ontology terms among positively selected genes (PSGs). (A) GO biological processes over-represented among PSGs in the Hexapoda lineage and (B) in the Collembola lineage. The size of the GO terms is proportional to the *p*-value obtained in the enrichment test [75], enriched terms were summarized and redundancy was removed with the REVIGO tool [76] using a semantic similarity threshold of 0.7 and *D*. *melanogaster* GO Database as reference. The complete dataset of GO enriched terms is presented in the S4 Table.

The Gene Ontology (GO) enrichment analysis of single-copy PSGs revealed that 68 biological processes (BPs), supported by 7 molecular functions (MFs), are enriched in the Hexapoda lineage (Fig 3A, S4 Table). These processes are predominantly involved in general metabolism, development (compound eye, ovarian follicle and general neurological development), biological regulation, transcriptional control and internal cell signaling. Among the enriched categories there is also DNA binding (damaged DNA binding, sequence-specific DNA binding), behavior (locomotor rhythm, circadian behavior), response to stimulus (response to other organisms, DNA repair, nucleotide-excision repair) and transport. Another 37 BPs are enriched within the multiple copy PSG clusters (Fig 3A, S4 Table) along the Hexapoda lineage. Most represented processes are associated with purine nucleotide metabolism, ATP metabolism and immune response. Other notable enriched categories are regulation of body fluids, development and ion homeostasis. Moreover, 16 MFs are enriched among the multiple copy PSG clusters along the Hexapoda lineage. Oxidoreductase activity (tRNA dihydrouridine synthase activity, monooxygenase activity) and binding are among them.

Over-representation analysis of GO terms associated with single-copy PSGs in the Collembola lineage revealed four significantly enriched BPs (Fig 3B, S4 Table). These BPs are involved in RNA metabolism and co-translational protein targeting. Ion homeostasis, ATP biosynthesis process, transport, ATPase activity, JAK-STAT signaling, binding (metal binding, coenzyme binding, drug binding) and oxidoreductase activity are part of 26 BPs and 10 MFs enriched within multiple-copy PSGs in Collembola.

The analysis of positive selection revealed genes and associated BPs and MFs that showed significantly accelerated evolution in the Hexapoda and Collembola lineages. A substantial number of the enriched categories in both lineages are associated with ATP metabolism and ATPases activity. Ge et al. [77] showed that ATPase genes could be involved in the adaptation to the new environments due to their important role in energy provision as shown in mammalian adaptive evolution. A particular function for some ATPases is ionic transport over the cell membrane, which we discuss below.

Ion homeostasis and ion transport are especially enriched among PSGs along the Collembolan branch and they are mainly represented by plasma membrane calcium-transporting Ca^2+^-ATPases and a sodium—chloride Na^+^-Cl^−^ cotransporter (S5 Table). Control of internal osmotic pressure at strict physiological values is important and such ion transporters need to be tightly regulated. In vertebrates, the transition from water to terrestrial ecosystems was accompanied by the acquisition of a strict hormone-mediated control of the sodium pump (Na^+^/K^+^-ATPase) and the sodium channel (ENaC) [78]. In hexapods, positive selection on genes participating in the regulation of body fluids could be related to adaptations to new osmotic pressure during the transition from water to soil. This may be particularly true for collembolan physiology, since water balance and osmoregulation are maintained in these animals by a specific organ called the ventral tube [12, 13].

DNA repair is identified to be under positive selection in the Hexapoda clade. Genes coding for ultraviolet (UV) excision repair protein Rad23, X-ray repair cross-complementing protein 6, an endonuclease III-like protein 1 and a replication protein represent this process (S5 Table). Evolution on land includes dealing with increased UV irradiation; this could explain the accelerated evolution of the DNA repair system. This was already shown in Tardigrades, which are invertebrates well known for their irradiation tolerance. These animals have evolved efficient UV repair systems among which Rad23 was identified [79]. Positive selection of the DNA repair system has also been observed in the Tibetan antelope. Recent sequencing of its genome revealed accelerated evolution of DNA repair-associated genes, suggesting that the adaptation to a high-altitude environment is characterized by resistance against high UV and low oxygen pressure [77]. Also, common tadpoles evolved tolerance along a gradient of increased UV irradiation, but the mechanism is yet unknown [80]. Furthermore, 26S proteasome non-ATPase regulatory subunit 8 was identified among the genes under positive selection in the Hexapoda clade. The 26S proteasome is involved in the regulation of repair mechanisms by assisting in disassembly of a DNA repair complex of protein that cannot be repaired adequately, but needs to be degraded [81].

Another stress factor to deal with is endogenous aromatic compounds as well as environmentally-derived xenobiotic compounds (organic pollutants, toxins, drugs), which are all being metabolized in the biotransformation pathway [82]. The most important enzyme families participating in this pathway are cytochrome p450s/monooxygenases, glutathione S-transferases and ATP-binding cassette (ABC) transporters. Monooxygenase activity was enriched among PSGs in the Hexapoda lineage and represented by monooxygenase and two cytochromes P450 (S5 Table). This protein family is well-known for its functional diversification in hexapods and associated with differences in life history and ecology of insect species [83]. From a functional perspective, monooxygenase activity is part of the first step in biotransformation (phase I), during which organic compounds are oxidized so that they become water-soluble. The adaptive evolution of the monooxygenases was linked to increased resistance to plant defense compounds and insecticides [83]. Moreover, one ABC transporter family was identified among the Hexapoda PSGs. Furthermore, two genes encoding heat shock proteins (Hsp70 and Hsp90), involved in more general stress response processes, were under positive selection in the Hexapoda lineage. Hsp70 and Hsp90 are chaperones that bind to proteins to assist folding. A more general feature of stress response is protein damage by unfolding. Hsp70 proteins immediately recognize damaged proteins and, in conjunction with E3 ubiquitin ligase, target damaged proteins to be recycled in the proteasome [84].

Immune response is another important target for positive selection, since this process is enriched among PSGs. Immune genes are under strong selective pressure to rapidly evolve against pathogens, and they are frequently found in large-scale analyses of positive selection in animals, both in deuterostomes [73, 85–87] and in protostomes [88]. More specifically, we identified a gene coding for protein croquemort among the immune response related genes under positive selection (S5 Table). Protein croquemort belongs to the CD36 receptor family, which is identified as macrophage receptor that recognizes apoptotic cells and subsequently mediates phagocytosis [89]. This gene was also identified to be a target of selection in a previous study involving genetic adaptation of stress tolerance in *O*. *cincta* [90]. Protein pellino belongs to the highly conserved toll-like receptor (TLR) family, which plays an important role in the innate immune response [91]. We note that TLRs are also under positive selection in mammals [92].

Furthermore, we identified substantial development-related processes to be under positive selection mostly along the Hexapoda lineage. They include chorion formation (outer egg membrane), neurological development (dendrite formation, mushroom body development, peripheral nervous system development), angiogenesis, ovarian follicle cell stalk formation, dorsal appendage formation, immune system development (lymph gland development), and compound eye/photoreceptor cell differentiation. These developmental processes could be important for the adaptation to new habitats.

Finally, we observe seven ribosomal proteins under positive selection in the Hexapoda lineage and six in the Collembola lineage. These genes were also identified to be under positive selection in an earlier study on the transition from land plants into aquatic environments [55]. As the ribosomal machinery is salt-sensitive, Wissler et al. suggested that the difference in osmotic pressure between aquatic and terrestrial environments could pose selective pressure on ribosomal genes [55].

## Conclusions

This study presents the first high-throughput next-generation sequencing and *de novo* assembly to characterize and annotate full transcriptomes of two collembolans, *F*. *candida* and *O*. *cincta*, which are both common model organisms in studies of soil ecology and genetic adaptation to stress [9, 93].

The comparative analysis of two springtail transcriptomes to three crustacean outgroup species and three insects revealed a range functional categories that likely were under strong selective pressure during divergence of Collembola and Hexapoda. These categories could be clustered in the following groups: genes involved in stress response against pathogens and toxic compounds; genes involved in interaction with the environment (i.e. osmotic pressure); genes involved in metabolism and more specifically energy metabolism; regulation and development-related processes. This study provides clues towards the toolkit used to adapt to terrestrialization and should be compared in the future to other terrestrialization events that occurred in the tree of life.

Alongside accelerated evolution of orthologous genes, we also show that Collembola contain many unique gene sets, which is comparable to the high number of orphan genes identified in other invertebrate species such as *D*. *pulex* and *A*. *pisum* [94] and the lone star tick *Amblyomma americanum* [95]. A variety of genetic mechanisms giving rise to new genes have been identified, including gene duplication, gene fusion and fission, exon shuffling, *de novo* origination from previously non-coding sequences and horizontal gene transfer [26]. Recently, we have identified horizontal gene transfer as a genetic mechanism that explains the presence of unique functional genes in collembolans [96]. The fact that *F*. *candida* is the first animal that is equipped with a biosynthesis pathway for β-lactam antibiotics [96] underpins the importance of such evolutionary processes in this animal group.

## Supporting Information

S1 Fig The distribution of contigs with length > 200 bp for *F*. *candida* and *O*. *cincta*. (TIF)

S2 Fig The distribution of Top BlastX hits for *F*. *candida* and *O*. *cincta* transcriptomes. (TIF)

S3 Fig iPATH 2.0. metabolic maps for *F*. *candida* (A) and *O*. *cincta* (B). (TIF)

S1 Table Summary of the NGS raw reads preprocessing for *F*. *candida* and *O*. *cincta*. (XLSX)

S2 Table OrthoMCL orthologous clusters shared among *A*. *pisum*, *D*.*pulex*, *F*. *candida*, *O*. *cincta*, *P*. *monodon*, *L*. *vannamei*, *P*. *humanus* and *T*. *castaneum*. The table indicates the number of proteins in the species assigned to the orthologous clusters.
(XLSX)

S3 Table The likelihood ratio test for the branch-site model with the foreground Hexapoda and Collembola branches in single-copy and multiple-copy gene clusters. (XLSX)

S4 Table Enriched biological processes and molecular functions among positively selected genes (PSGs) in the Hexapoda and in the Collembola lineages. (XLSX)

S5 Table Gene descriptions associated with enriched biological processes and molecular functions among PSGs in the Hexapoda and in the Collembola lineages. (XLSX)
