# Supplementary figures and images for: Collembolan Transcriptomes Highlight Molecular Evolution of Hexapods and Provide Clues on the Adaptation to Terrestrial Life
Source: PLoS One. 2015 Jun 15;10(6):e0130600. doi: 10.1371/journal.pone.0130600 (PMC4468109; doi:10.1371/journal.pone.0130600)

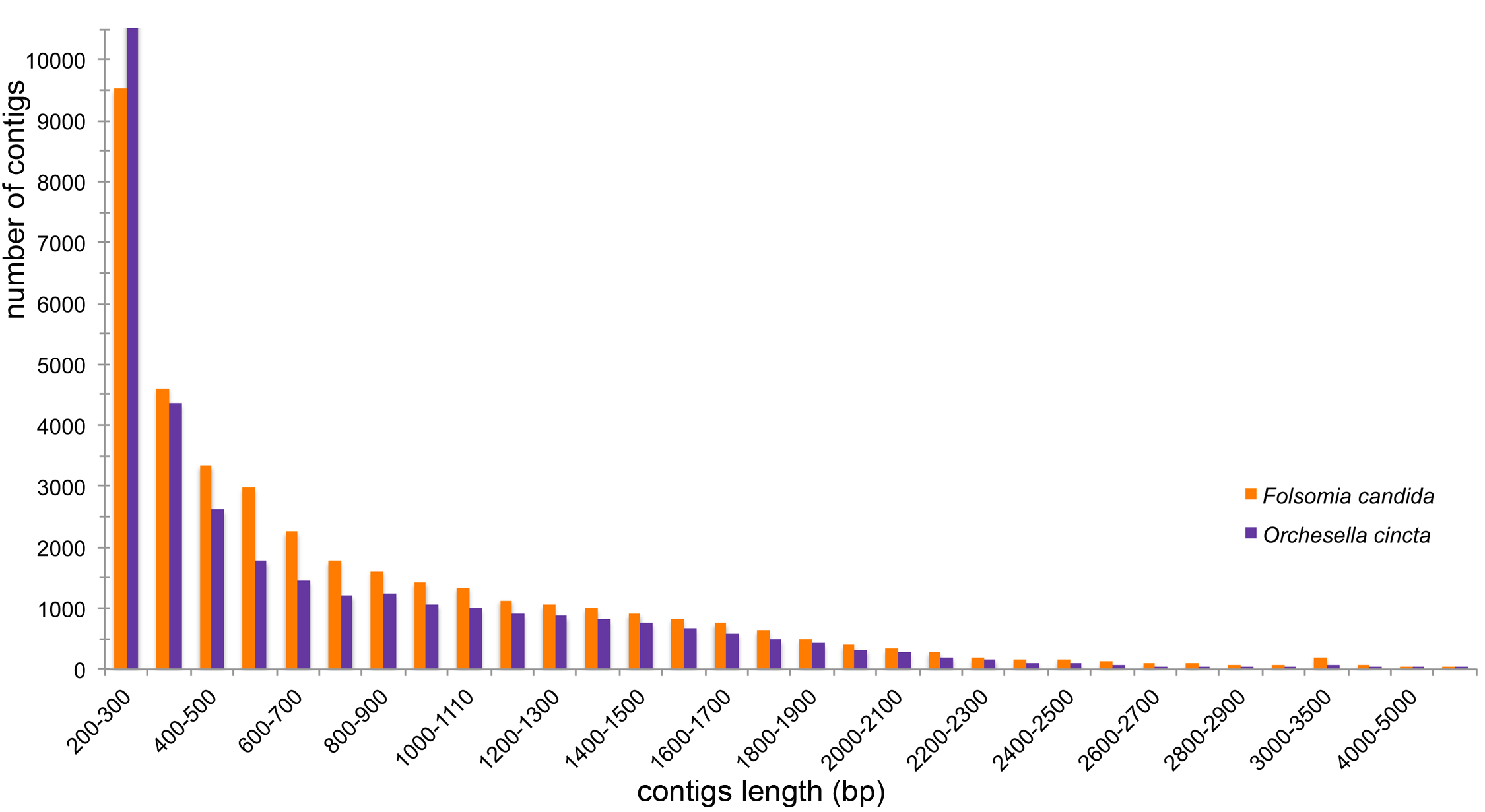

Supplement: S1 Fig — (TIF) [file pone.0130600.s001.tif]

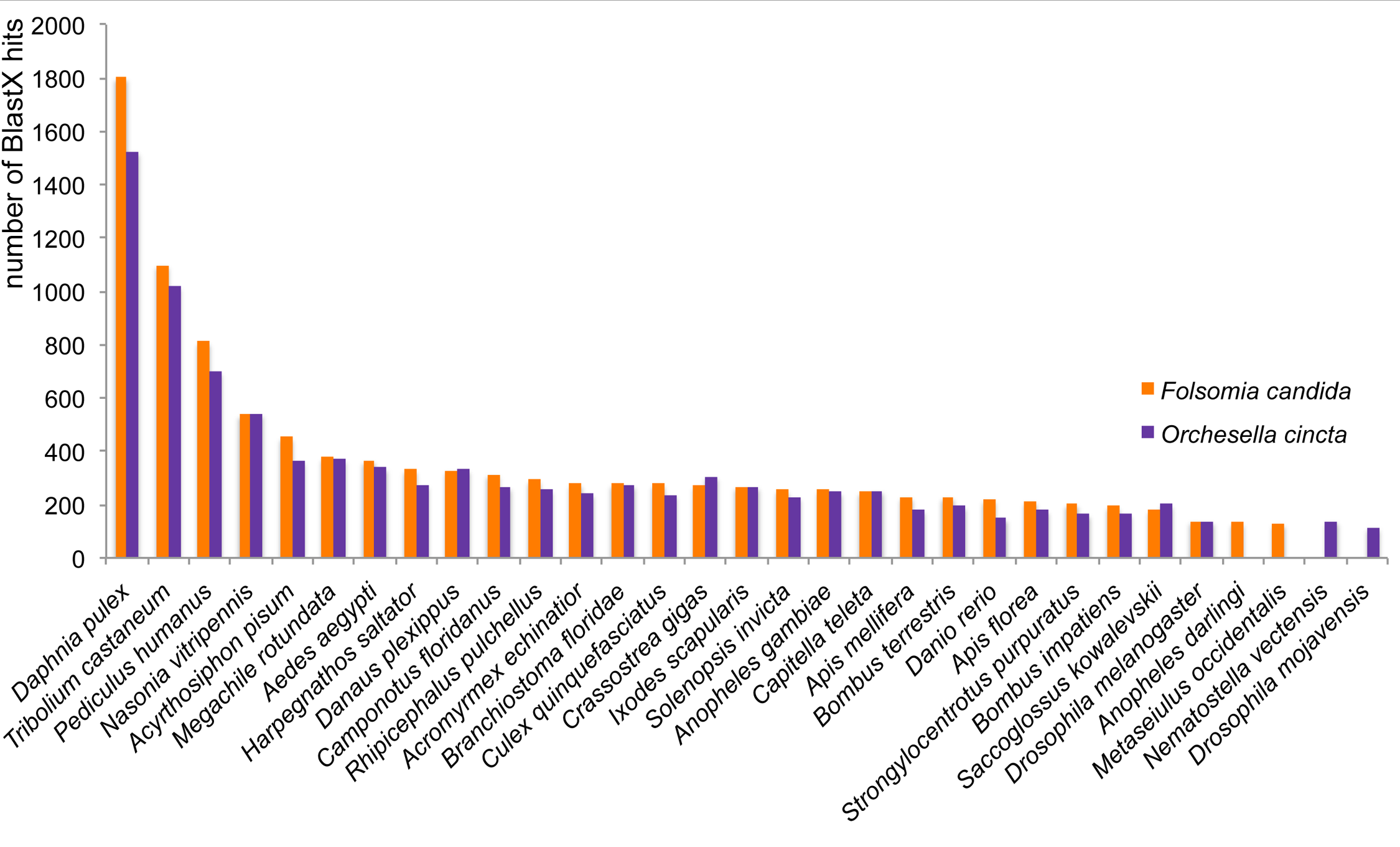

Supplement: S2 Fig — (TIF) [file pone.0130600.s002.tif]

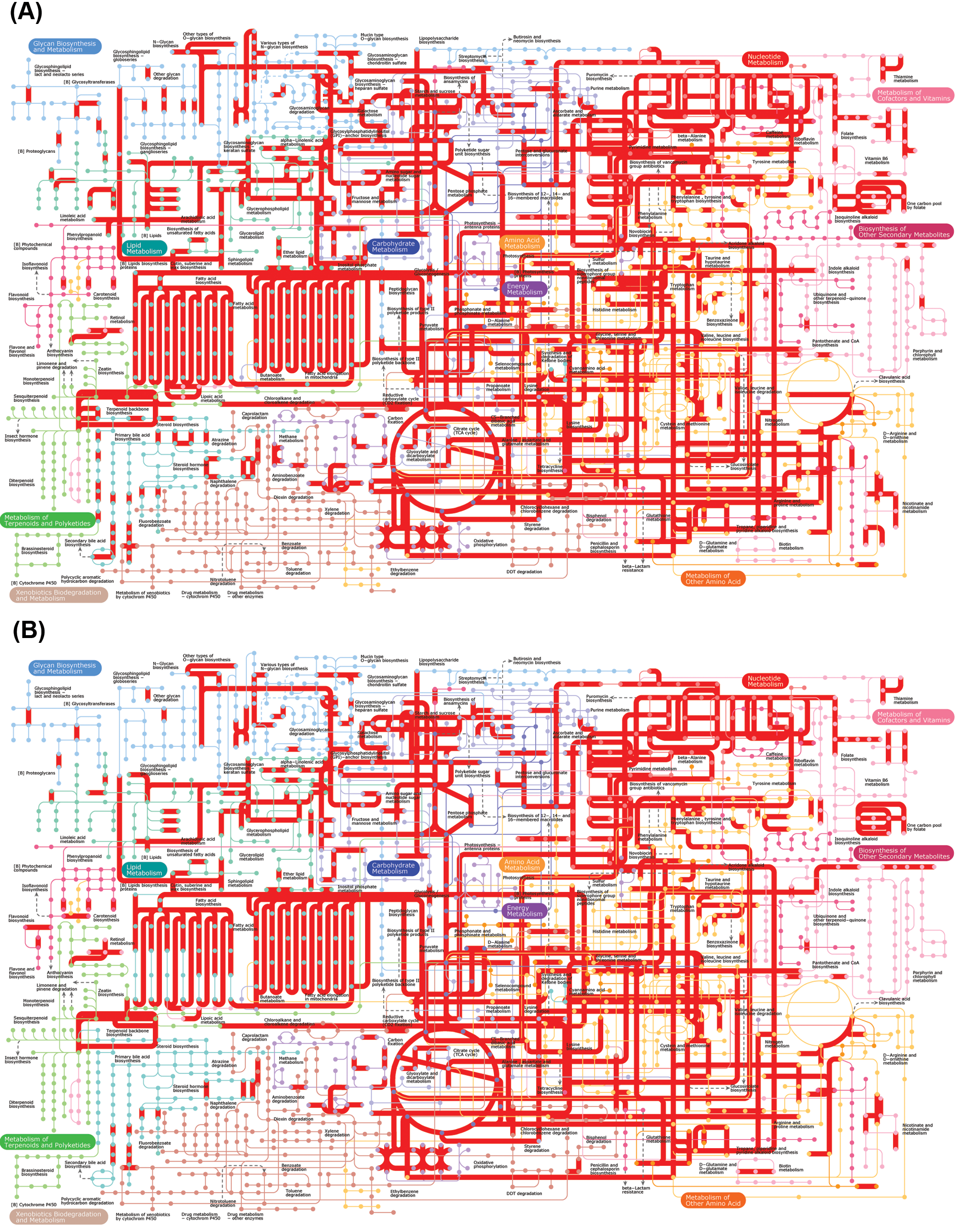

Supplement: S3 Fig — (TIF) [file pone.0130600.s003.tif]
